# Interleukin-24 in type 2 immune diseases

**DOI:** 10.3389/fimmu.2026.1776623

**Published:** 2026-03-12

**Authors:** Xiaoting Song, Dong Lan, Fang Liu

**Affiliations:** 1 Department of Dermatology, Beijing Chaoyang Hospital, Capital Medical University, Beijing, China; 2 Department of Dermatology, Jingxi Campus, Beijing Chaoyang Hospital, Capital Medical University, Beijing, China

**Keywords:** atopic allergic diseases, IL-24 cytokine, JAK - STAT signaling pathway, targeted therapy, type 2 immune diseases

## Abstract

Interleukin (IL)-24, a member of the IL-20 cytokine family, is secreted by multiple cell types, such as immune cells (T cells, B cells, NK cells, macrophages), and non-immune cells (epithelial cells and fibroblasts). IL-24 and its downstream signaling pathways mediate vital biological processes, including processes governing cell growth, fate determination, cell death, and inflammation, albeit with effects that are context-dependent across disease states. In this review, we present comprehensive summary and recent breakthroughs in IL-24 characterization and its emerging pathogenic functions in type 2 immune diseases, including chronic spontaneous urticaria (CSU), atopic dermatitis (AD), allergic contact dermatitis (ACD), bullous pemphigoid (BP), chronic nodular prurigo (CNPG), and allergic airway diseases, in an attempt to provide valuable insights for developing its potential as biomarkers or therapeutic targets.

## 1 Introduction

Type 2 immune diseases comprise chronic allergic and atopic conditions that arise at epithelial barrier sites—including the skin, airways, and gastrointestinal tract. These disorders are characterized by (1) a predominant type 2 inflammatory response driven by IL-4, IL-5, and IL-13; (2) the involvement of key effector cells including Th2 cells, type 2 innate lymphoid cells (ILC2s), eosinophils, and mast cells; and (3) elevated levels of type 2-associated biomarkers such as IgE and periostin (1).

IL-24, a cytokine in the IL-20 family, was first discovered in human melanoma cells following treatment with interferon-β (IFN-β) and mezerein (a protein kinase C activator) in 1996, receiving its initial name as the melanoma differentiation-associated (MDA)-7 antigen (2). It shares 69% homology with mouse FISP (3) and 78% with rat C49a/mob-5 (4). It was subsequently renamed IL-24, following recognition that it functions as a multifunctional cytokine within the IL-20 family (a subset of the IL-10 family), which includes IL-19, IL-20, IL-22, IL-24, and IL-26 (5). Early investigations revealed that IL-24 primarily exerts inhibitory effects on the proliferation of various tumor types, thereby conferring extensive anticancer properties. Beyond its antitumor effects, IL-24 engages in diverse physiological processes—tissue repair and remodeling, immune and inflammatory regulation, and host homeostatic balance—and critically contributes to autoimmune and inflammatory pathogenesis, particularly in type 2 immune diseases. In this review, we focus on the pathogenesis of IL-24 in various type 2 immune disorders, such as chronic spontaneous urticaria (CSU), atopic dermatitis (AD), allergic contact dermatitis (ACD), bullous pemphigoid (BP), chronic nodular prurigo (CNPG), and allergic airway diseases, and provide a synopsis of recent advances in IL-24- and receptor-targeted clinical investigations, thereby offering new perspectives for the development of potential therapeutic strategies.

## 2 Source and target cells of IL-24

**Figure 1:**
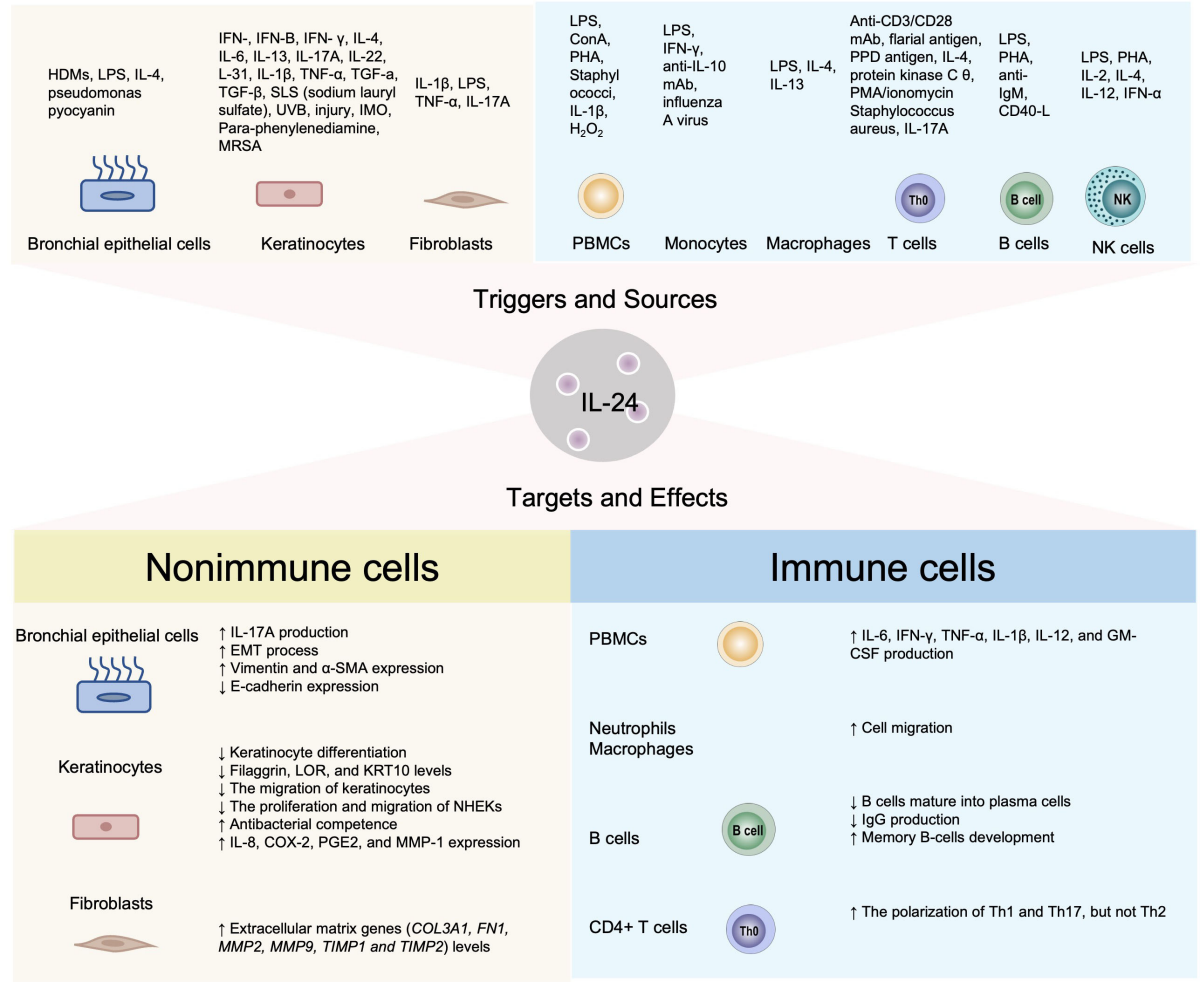
Cell resources and effects of IL-24.

IL-24 can be synthesized by and function on both immune cells (T cells, and B cells, NK cells, macrophages) and non-immune cells (keratinocytes, bronchial epithelial cells, fibroblasts and tumor cells) in various situations. Compared with activated T cells, monocytes/macrophages, and epithelial cells, B cells and NK cells constitute secondary or minor sources and responders of IL-24, with their participation being largely confined to specific experimental settings or disease contexts rather than representing a dominant biological role. IL-24 is known to be upregulated by a spectrum of cytokines encompassing Th1 (IFN-γ), Th2 (IL-4, IL-13, IL-31), Th17 (IL-17A), Th22 (IL-22), and pro-inflammatory cytokines (IL-1β, IL-6, TNF-α) (**Figure 1**) (6). Among these inducers, the effect of type 2 cytokines on IL-24 generation has been extensively investigated. Spontaneous development of chronic inflammatory skin lesions with persistent pruritus was observed in IL-4 transgenic mice, which exhibited IL-24 expression levels several hundred times higher than wild-type controls (7). Through chromatin immunoprecipitation sequencing (ChIP-seq), Wei et al. demonstrated robust STAT6-mediated activation of IL-24 in Th2 cells (8). Similarly, STAT6-dependent IL-24 generation also occurs in keratinocytes and bronchial epithelial cells (9). However, in mitogen-stimulated PBMCs, IL-24 is selectively induced by IL-2, IL-7, IL-15, TNF-α, GM-CSF, and IL-1β, but not by interferons or Th2 cytokines (10). The cellular sources and regulatory stimuli for IL-24 are summarized in **Figure 1**.

### 2.1 T cells

IL-24 serves as a functional mediator in type 2 immune responses, which is principally derived from activated Th2 cells (3). Studies demonstrate that IL-24 expression in Th2 cells requires both TCR-PKC and IL-4R-STAT6 signaling pathways (3). STAT6 activation of the IL-24 gene in Th2 cells has been established through ChIP-seq evidence (8). In this process, c-Jun partners with STAT6 to orchestrate IL-24 promoter binding and gene expression in Th2 cells (11). In type 2 immune responses, IL-24 play a immunomodulatory role, through inhibiting IFN-γ-producing Th1 and IL-17A-producing Th17 cells, which has been documented in healthy naive CD4+ T cell models (12), lymphatic filariasis (13), pulmonary tuberculosis (14).

Besides its well-characterized production by Th2 cells, IL-24 can also be generated by Th17 and Th9 cells and can modulate their function. IL-24, upregulated by IL-17A/NF-κB signaling, has been demonstrated to attenuate Th17-associated inflammatory responses and confer protection against autoimmune uveitis in experimental models (15). IL-24 expression in Th9 cells is enhanced by pre-exposure to *Staphylococcus aureus* (*S. aureus*) *(*16).

### 2.2 B cells

Within human follicular B cells, IL-24 is predominantly expressed by CD27 positive memory and CD5 positive B cells, whereas centroblasts and plasma cells show minimal to no expression (17). Peripheral B cells from patients with active inflammatory bowel disease (IBD) are reported to produce IL-24 (18). IL-24 elicits context-dependent, paradoxical effects on B cell biology. In the presence of CD40L, IL-24 enhances B cell proliferation while suppressing plasma cell differentiation and antibody production (17). Conversely, in CD40L-naive conditions, IL-24 induces apoptosis in human B cells by initially triggering cell cycle arrest, followed by the activation of the mitochondrial pathway at a later stage (19).

### 2.3 NK cells

NK cells serve as a functional link between innate and adaptive immune arms (20). IL-24 expression in murine NK cells is triggered by type 1 interferon through the STAT6 pathway (21) and by mitogenic stimuli (10). The effects of IL-24 on NK cells are controversial, possibly due to differences in source/format, functional outcomes, and disease background. IL-24 from decidual stromal cells facilitates NK cell differentiation (22), whereas recombinant IL-24 fails to influence NK cell activation or migration in human NK-92 cell line (23).

### 2.4 Macrophages

Macrophages were the primary source of IL-24 expression, along with T cells, in mitogen-stimulated PBMC cultures (10). Macrophage-derived IL-24 is upregulated by LPS and IL-4 via STAT6 (21, 24). In mammary tumors, IL-24 expression was induced in macrophages *in vitro* by co-stimulation with IL-4 and TLR4 agonists (25). Conversely, IL-24 could act solely or in concert with IL-4 to promote macrophage M2 polarization through the same pathway (26, 27). IL-24 promotes the migration of human monocytes *in vitro* and orchestrates myeloid cell chemotaxis *in vivo (*28).

### 2.5 Epithelial cells

Keratinocytes, specifically the CD45-Krt10^+^ subset, were shown to increase IL-24 production under conditions of para-phenylenediamine (PPD)-mediated contact hypersensitivity (29). In an experimental murine dermal wound model, IL-24 is specifically upregulated in wound-edge epithelial stem cells under the control of hypoxia and STAT3 (30). *In vitro* cell arrays, IL-24 production by keratinocytes and bronchial epithelial cells is induced by IL-13 through STAT6 signaling (9).

### 2.6 Fibroblasts

IL-24 has been detected in gingival fibroblast cells (31), colon fibroblasts (32, 33), synovial myofibroblasts (34). A WNT5A+/IL24+ fibroblast state was identified in skin lesions of psoriasis, and signals from WNT5A+/IL24+ fibroblasts have been shown in in-silico and *in-vitro* studies to upregulate multiple inflammatory genes in keratinocytes (35). Single-cell RNA sequencing identified IL-24 as a highly upregulated gene shared between keratinocytes and fibroblasts in PM-exposed ex vivo skin (36).

## 3 The IL-24 downstream signaling pathways

**Figure 2:**
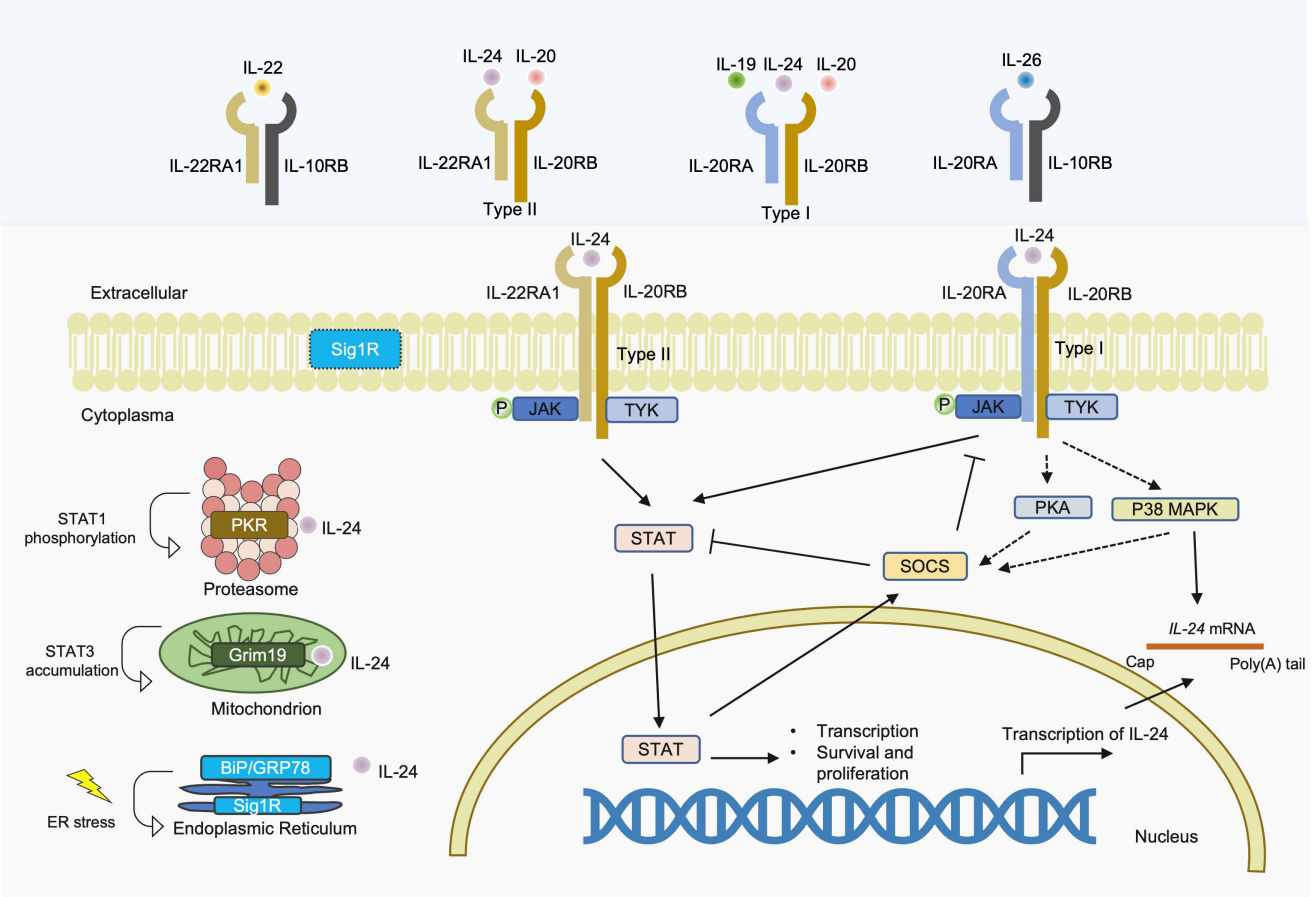
IL-24 signaling pathways and shared receptor subunits.

IL-24 can interact with receptors in the cell membrane, endoplasmic reticulum (ER), cytoplasm, and mitochondria (**Figure 2**). Canonical IL-24 signaling proceeds via sequential activation events: plasma membrane IL-20 receptor engagement and downstream cytoplasmic Janus protein tyrosine kinases (JAK) and signal transducers and activators of transcription (STAT) cascade induction.

The noncanonical, JAK/STAT-independent arms of IL-24 signaling recruit distinct cellular machinery: cytosolic protein kinase R (PKR), mitochondrial respiratory chain proteins, and ER chaperones exemplified by sigma 1 receptor (Sig1R).

### 3.1 JAK/STAT-dependent signaling pathway

IL-24 signals through two distinct heterodimeric receptor complexes: the type I receptor (IL-20RA/IL-20RB), shared with IL-19 and IL-20, and the type II receptor (IL-22RA1/IL-20RB), shared with IL-20. The three receptor subunits are all expressed on non-immune cells, such as keratinocytes and bronchial epithelial cells, while IL-20RB is also expressed on immune cells. In different tissues, while IL-22RA1 is highly expressed in epithelial-rich tissues including the skin, intestine, liver, kidneys, and pancreas, IL-20RA exhibit a broader but distinct distribution pattern with robust expression in the skin, lungs, and reproductive organs (ovary, testes, placenta) and notably low levels in the intestine and liver (37). Specifically, in keratinocytes, the expression of IL-22RA1 was approximately 10 times higher than that of IL-20RA (38), suggesting that IL-20 and IL-24 primarily act on these cells via the type II receptor complex. Compared to IL-22RA1, which is largely restricted to epithelial cells, IL-20RA shows broader expression, including low-level constitutive expression on monocytes and dendritic cells (39, 40) and can be further induced in macrophages under inflammatory conditions (41). This differential expression pattern suggests that IL-24 signaling through IL-22RA1/IL-20RB predominantly affects epithelial homeostasis, while IL-20RA/IL-20RB signaling may modulate immune cell function in inflammatory contexts. IL-22, in contrast, exclusively uses the IL-22RA1/IL-10RB complex. Despite sharing the IL-22RA1 receptor subunit, IL-24 and IL-22 exhibit fundamentally distinct cellular sources and modes of action. IL-24 originates from ubiquitous epithelial expression, ensuring broad autocrine signaling. Conversely, IL-22 is mainly synthesized by immune cells (T cells and innate lymphoid cells) that penetrate the epidermis for focused, proximity-dependent delivery (42). IL-26 signals through IL-10RB/IL-20RA. Although these cytokines share receptor subunits and exhibit functional overlap in STAT3 activation and epithelial responses, emerging evidence indicates context-specific, non-redundant roles: immune activation for IL-19, skin homeostasis for IL-20, and tumor apoptosis and terminal keratinocyte differentiation inhibition for IL-24, whereas IL-22 promotes epithelial proliferation, IL-26 exhibits unique antimicrobial properties (41, 43).

Upon binding, these receptors trigger the JAK-STAT pathway. This cascade encompasses the activation of JAK1, JAK3, and TYK2, which subsequently drives downstream phosphorylation of STAT1 and STAT3 (44). IL-24 activates both STAT1 and STAT3 through either receptor complex (44), with STAT3 activation occurring at low physiological ligand concentrations and STAT1 activation requiring substantially higher levels (41). Furthermore, via the JAK/STAT-dependent activation of SOCS1 and SOCS3, IL-24 could attenuate the inflammatory Th17 cytokine program, thereby reducing Th17 cell pathogenicity (15).

### 3.2 Noncanonical JAK/STAT-independent signaling pathways

IL-24 stimulation leads to activation of various receptor-dependent intracellular signaling cascades, including activation of PKA and p38 pathway (40), which can also further induce JAK/STAT-independent SOCS protein activation and stabilize IL-24 mRNA (45, 46). Within the ER, IL-24 engaged the molecular chaperones BiP/GRP78 (47) and Sig1R (48), leading to ER stress. At the inner mitochondrial membrane, IL-24 interacts with respiratory chain protein Grim19, driving STAT3 accumulation within mitochondria (49). In the cytosol, IL-24 also can interact with PKR, thereby inducing STAT1 phosphorylation (50).

## 4 The pathological role of IL-24 in allergic diseases

While IL-24 is broadly expressed, its major cellular sources are disease-specific. Th2 cells are common across these allergic diseases, yet distinct patterns predominate: keratinocytes (AD, BP, ACD), mast cells (CSU), fibroblasts (CNPG), and bronchial epithelial cells (airway diseases).

### 4.1 Chronic spontaneous urticaria

As a mast cell-driven disease, CSU encompasses autoimmune endotypes involving mast cell activation by IgE and/or IgG autoantibodies (51). IL-24 was identified by Schmetzer et al. as a common and specific autoantigen for IgE in CSU patients (52). Furthermore, it was shown that elevated IL-24 expression corresponds to greater disease severity in CSU (53). The immunological impact of autologous serum therapy encompasses a decline in IgE antibodies against IL-24 (53). In CSU patients, IL-24 is detectable in spontaneous urticarial wheals, with elevated gene and protein expression in PBMCs from a patient subset (54). *In vitro* studies have demonstrated that IL-24 induces histamine release from human mast cells sensitized with IgE purified from CSU patients, whereas control cells remain unresponsive (52). Moreover, it is reported that T cell-derived microvesicles induce mast cell production of IL-24, which suggests a role of T cell-mast cell-IL24 axis in disease pathogenesis (55). These results indicate that targeting IL-24 represents a fundamental mechanism underlying CSU treatment.

### 4.2 Atopic dermatitis

AD is a multifaceted condition with complex pathogenic processes including genetics, skin barrier dysfunction, skin microbiota disorder, and immune dysregulation. Experiments in normal human epidermal keratinocytes have shown that the IL-13/periostin/IL-24 signaling cascade disrupts epidermal barrier function by downregulating filaggrin expression in allergic skin inflammation (9). In IL-31-stimulated keratinocytes, IL-24, together with IL-20, downregulate the expression of filaggrin (56). Moreover, siRNA-mediated knockdown of IL-24 or its downstream effector STAT3 could enhance aryl hydrocarbon receptor (AHR) modulator-induced upregulation of filaggrin and loricrin, suggesting that IL-24/STAT3 axis inhibition may improves skin barrier dysfunction (57). Recently, Penta-O-Galloyl-b-D-Glucose (PGG), discovered by in silico screening, functions as an IL-24 signaling inhibitor that improves skin barrier function through STAT3 suppression (58).

As previously noted, Myles et al. discovered that IL-24 inhibits IL-1β expression in keratinocytes, which subsequently leads to decreased IL-17A expression in γδ T cells and increased neutrophil infiltration in the skin (59), which may facilitate the colonization of *Staphylococcus aureus* (*S. aureus*) in AD skin. In addition, IL-24 can promote type 2 immunity via the JAK-STAT-IL-33 axis in AD, thereby fueling methicillin-resistant *S. aureus* (MRSA)-induced AD-like inflammation (60).

AD is a highly heterogeneous inflammatory skin disease and can be classified as different endotypes based on the molecular profiling. Asian AD and pediatric AD patients show a Th17/Th2 or blended AD-psoriasis endotype (61). IL-24 has been reported to be upregulated in psoriatic epidermis and activated its downstream STAT3 triggered psoriasis-like skin inflammation in mice (62). In the keratin 5-driven IL-24 transgenic mouse model, IL-24 overexpression results in neonatal lethality and a psoriasiform phenotype characterized by marked epidermal hyperplasia with impaired keratinocyte differentiation, accompanied by prominent dermal infiltration of macrophages—consistent with the observation that keratinocyte-derived MCP-1 drives macrophage recruitment restricted to the dermal layer in human psoriasis (63). Therefore, these results suggest IL-24 involvement in Th2/Th17 or blended AD-psoriasis endotype of AD.

We performed a bioinformatics analysis of IL-24 expression using the publicly available human transcriptomic dataset GSE130588, which comprises 208 samples: normal skin (n=20), pre-treatment lesional skin (preL, n=51), pre-treatment non-lesional skin (preNL, n=42), post-treatment lesional skin (postL, n=73), and post-treatment non-lesional skin (postNL, n=22). Using unpaired t-tests, we observed that IL-24 expression was significantly upregulated in lesional skin compared with non-lesional skin prior to treatment (*p* < 0.0001) and significantly downregulated in lesional skin following dupilumab treatment (*p* < 0.01) (**Supplementary Figure 1**). These findings suggest the important role of IL-24 in AD pathogenesis and its response to treatment.

Taken together, IL-24 is crucial for barrier dysfunction that occurs in type 2 inflammation, facilitates *S. aureus* colonization, and triggers psoriasis-like inflammatory responses in the pathogenesis of AD. However, studies specifically quantifying IL-24 and its correlation with disease severity scores in AD patients remain to be conducted.

### 4.3 Allergic contact dermatitis

Para-Phenylenediamine (PPD) is a common contact allergen used in hair dyes. In PPD allergic patients, IL-24 levels are elevated in affected skin compared to uninvolved areas, and the increased expression correlates positively with the severity of clinical symptoms (29). In mouse models, IL-22RA1, IL-20RB, and IL-24 knockout show partial resistance to PPD-induced contact hypersensitivity (29). In addition, in a mouse model of ACD induced by topical application of 2,4-dinitrofluorobenzene (DNFB), elevated expression of IL-24 has also been observed (38).

In keratinocyte-specific conditional knockout mice, by attenuating *Il24* gene expression in keratinocytes, HMGB1-mediated chromatin remodeling confers protection from ACD (64). However, in a T cell-dependent contact hypersensitivity model, IL-20RB-deficient mice exhibited increased sensitivity to the contact allergen trinitrochlorobenzene (65), suggesting IL-20RB signaling dampens antigen-specific T cell activation. These conflicting findings may be explained by several factors: (1) allergen characteristics, with para-phenylenediamine being a hapten-type allergen requiring metabolic activation versus direct sensitizers; (2) differential receptor engagement, with IL-22RA1/IL-20RB signaling in epithelial keratinocytes versus IL-20RB on T cells potentially modulating distinct phases of the hypersensitivity response; and (3) context-dependent IL-24 function, which can exert either pro-inflammatory or regulatory effects depending on the predominant cellular target. The role of IL-24 and its receptors in different allergen mediated contact dermatitis remains to be further explored.

### 4.4 Bullous pemphigoid

Bullous pemphigoid (BP) is the most common autoimmune bullous disorder, driven by type 2 inflammation and immunity. Elevated IL-24 has been observed in BP patients, correlating with more severe disease (66). Moreover, IL-24-treated HaCaT cells show reduced BP180 levels, suggesting that IL-24 may disrupt keratinocyte function and contribute to BP by modulating proteins crucial for skin integrity (66). In addition, IL-24 was one of upregulated cytokines when primary keratinocytes were stimulated with IgG from BP patients (67), while the role of IL-24 identified in BP-IgG-treated keratinocytes warrants further investigation.

### 4.5 Chronic nodular prurigo

CNPG presents clinically as hyperkeratotic nodules with severe pruritus, predominantly affecting the extremities and trunk. A study profiled CNPG using single-cell RNA sequencing and found that, compared with AD and healthy controls, CNPG exhibited markers associated with extracellular matrix remodeling, collagen production, and fibrosis, featuring a distinct group of CXCL14-IL24+ secretory papillary fibroblasts (68).

### 4.6 Allergic airway diseases

Type 2 immune inflammation and tissue damage pathways are common to allergic airway diseases such as allergic rhinitis and asthma. In patients with allergic asthma, sputum IL-24 was increased during the allergic asthma season, and elevated nasal IL-24 was correlated with poor asthma control scores (69). Flow cytometric analysis of sputum revealed elevated IL-24 was correlated inversely with Treg cells (69). Experiments in primary normal human bronchial epithelial cells showed that IL-24 expression was induced by IL-4 stimulation (70). These findings suggest IL-24 might act as a representative nasal biomarker for allergic airway diseases.

## 5 Therapeutic application

IL-24 plays a crucial role in the pathophysiology of type 2 immune diseases. Therapeutic strategies targeting IL-24, its receptor complexes, or downstream signaling molecules hold promising potential. Up to now, there have been limited clinical studies on the treatment of type 2 immune diseases by targeting IL-24.

The IL-22RA1 monoclonal antibody ARGX-112 (temtokibart) has shown considerable therapeutic potential and is currently in phase II clinical trials for patients with moderate to severe AD. Phase I (NCT05099133, NCT03514511), phase IIa (NCT04922021, NCT05470114, EUCTR2020–005541-16-DE) have completed and demonstrated its safety and tolerability. The phase IIa trial results (NCT04922021) in 58 adults demonstrated that temtokibart achieved significant clinical improvement in AD (71). By week 16, the temtokibart arm showed a mean EASI decrease of 15.3 points (65.4% improvement) compared with 3.5 points (19.7% improvement) in the placebo group (*p* = 0.003). Response rates favoring temtokibart included EASI-75 (41.6% vs 13.7%; *p* = 0.011), EASI-90 (30.8% vs 3.5%; *p* = 0.003), and EASI-100 (20.9% vs 0%; *p* = 0.006). Temtokibart therapy over 16 weeks yielded a significant 65.4% improvement in the primary efficacy endpoint (EASI), an effect size comparable to dupilumab at similar development stages (72)and surpassing that of experimental OX-40 pathway antagonists (73). IL-22RA1 blockade reverses histopathological and molecular aberrations underlying AD across *in vitro*, ex vivo, and *in vivo* model systems (42), which indicated that targeting IL-22RA1 ameliorates clinical symptoms through multiple pathways, including modulation of immune inflammation and restoration of skin barrier function. The results from the phase IIb trial (NCT05923099) in 262 adult patients with AD showed that investigational agent temtokibart met its primary endpoint and well-tolerated at week16, that is, it achieved percentage change in the eczema area and severity index (EASI) compared with placebo (−41.7%) for the three doses treatment groups (600 mg, −61.2%, *p*<0.01; 450 mg, −57.1%, *p*<0.05; 300 mg, −64.3%, *p*<0.01) (74, 75). The pooled data also demonstrated that temtokibart-treated patients (n=22 at baseline, n=14 at week 16) achieved a 97% improvement in immune gene expression and significant restoration of epidermal barrier-related gene expression by week 16 (76).

Temtokibart targets the IL-22RA1 subunit shared by both the IL-22 receptor (IL-10RB/IL-22RA1) and the type II receptor (IL-20RB/IL-22RA1). Consequently, its therapeutic efficacy likely reflects dual blockade of IL-22 and IL-24 signaling rather than selective IL-22 inhibition alone. This simultaneous interference with IL-20 and IL-24 pathways via the IL-20RB/IL-22RA1 heterodimer likely underlies the enhanced efficacy observed with IL-22RA1-directed therapy compared with exclusive IL-22 inhibition (71, 77, 78).

## 6 Conclusion and future perspectives

This review offers a comprehensive look at the latest research exploring the involvement of IL-24 and its downstream signaling pathways in type 2 immune diseases, suggests its potential as a therapeutic candidate for type 2 immune diseases. IL-24 initiates its biological effects by interacting with its specific receptors, a process that may engage the JAK/STAT signaling cascade or operate via alternative pathways. The concentration of IL-24 has been shown to correlate with the severity of some type 2 immune diseases and responses to treatment, suggesting its potential utility as a predictive biomarker for disease trajectory and treatment outcomes.

Although considerable progress has been made, these blind spots continue to obscure the full picture of IL-24’s role in type 2 immunity: (1) heterogeneous experimental designs and disease models that frustrate cross-study and cross-disease comparisons; (2) an incomplete map of how IL-24 intersects with broader cytokine networks; and (3) the absence of longitudinal data tracking IL-24 dynamics throughout disease progression. Due to the current limitations in available data, additional research is crucial to elucidate the exact roles and underlying mechanisms of IL-24 in these areas. At present, exploration of therapeutic application targeting IL-24 and its receptors in type 2 immune diseases remains in its early stages.

## Supplementary material

Supplementary Figure 1 Analysis of IL-24 expression using the publicly available GSE130588 dataset. Normal skin (n=20), preL, pre-treatment lesional skin (n=51), preNL, pre-treatment non-lesional skin (n=42), postL, post-treatment lesional skin (n=73), postNL, post-treatment non-lesional skin (n=22).
