# Supplementary material for: Interleukin-24 in type 2 immune diseases
Source: Front Immunol. 2026 Mar 12;17:1776623. doi: 10.3389/fimmu.2026.1776623 (PMC13017265; doi:10.3389/fimmu.2026.1776623)
Supplement: Supplementary Figure 1 — Analysis of IL-24 expression using the publicly available GSE130588 dataset. Normal skin (n=20), preL, pre-treatment lesional skin (n=51), preNL, pre-treatment non-lesional skin (n=42), postL, post-treatment lesional skin (n=73), postNL, post-treatment non-lesional skin (n=22). [file DataSheet1.docx]

**Supplementary Material**


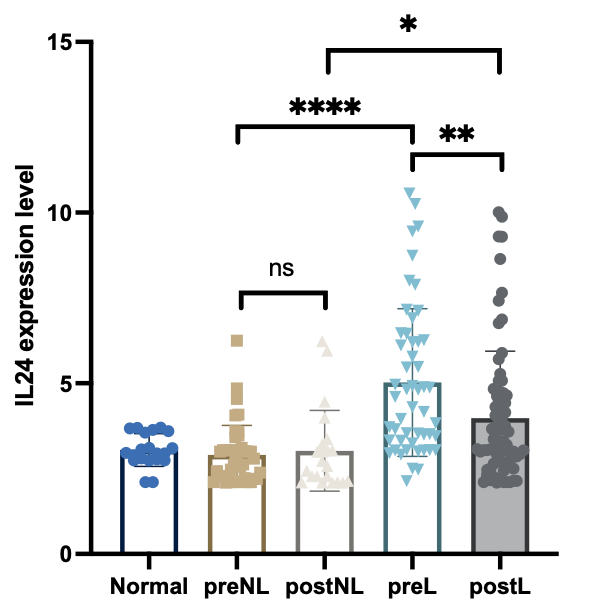


**Figure 1.** Analysis of IL-24 expression using the publicly available GSE130588 dataset.

Normal skin (n=20), preL, pre-treatment lesional skin (n=51), preNL, pre-treatment non-lesional skin (n=42), postL, post-treatment lesional skin (n=73), postNL, post-treatment non-lesional skin (n=22).
